# A Case Report of a Rapidly Progressive Epstein-Barr Virus Encephalitis Infection in an Adult With HIV on Highly Active Antiretroviral Therapy

**DOI:** 10.7759/cureus.52392

**Published:** 2024-01-16

**Authors:** Dunstan A Akolbire, Doris Akolbire, Robert Delapenha

**Affiliations:** 1 Internal Medicine, Sovah Health Hospital, Martinsville, USA; 2 Internal Medicine, Howard University Hospital, Washington, USA; 3 Infectious Disease, Howard University Hospital, Washington, USA

**Keywords:** csf diagnosed ebv, antiviral therapy in ebv, acyclovir in ebv encephalitis, ebv in hiv positive, ebv encephalitis on haart

## Abstract

Epstein-Barr virus (EBV) encephalitis is a rare complication of EBV infection, with most cases described in children. Although some cases of EBV encephalitis have been reported in adults, they have occurred in the presence of other central nervous system infections, superimposed on an underlying neurocognitive disorder, or in immunocompromised states. We present herein a rare case of rapidly progressive EBV encephalitis in an adult male with HIV infection on highly active antiretroviral therapy (HAART) with no pre-existing neurocognitive symptoms. A 52-year-old African American man with HIV infection on HAART presented with acute altered mental status and weakness. On admission, he had normal muscle tone and reflexes, with no signs of meningism. Head CT without contrast showed no acute intracranial pathology. Blood and urine cultures were negative. CSF analysis was suggestive of a viral infection. Viral studies were positive only for EBV DNA by PCR in CSF. The patient received IV acyclovir for two weeks, followed by four weeks of oral valacyclovir with full recovery. Clinicians should consider a diagnosis of EBV encephalitis in HIV-positive patients on HAART who present with acute altered mental status. Treatment with antiviral therapy should be considered in patients with EBV encephalitis.

## Introduction

Epstein-Barr virus (EBV) is a DNA lymphotropic herpesvirus that is highly prevalent and infects about 90% of the human population worldwide [1-3]. Primary infection with the virus is usually asymptomatic and progresses to latency in healthy individuals [3,4]. In some cases, EBV infection manifests acutely as Infectious Mononucleosis (IM) in adolescents, characterized by pharyngitis, cervical lymphadenopathy, fever, and fatigue [5]. Rarely, it may present with neurological manifestations like encephalitis, aseptic meningitis, transverse myelitis, acute cerebellar ataxia, and acute demyelinating encephalomyelitis [6-9].

EBV has also been noted in cerebrospinal fluid (CSF) with an unknown significance, but the diagnosis of EBV encephalitis can be made when viral encephalitis is clinically suspected with evidence of EBV in CSF in the absence of other causative agents [10]. The pathophysiology of these neurological manifestations is unclear, but there have been theories of neuronal infiltration by CD8-positive cells and antigen-antibody complex deposition leading to endothelial damage and tissue injury [11].

While there have been a few reports of primary EBV infection presenting with neurological signs in children, this is uncommon [6,12]. Although some cases of EBV encephalitis have been reported in adults, these have occurred during coinfections with other central nervous system infections, superimposed on an underlying neurocognitive disorder, or in immunocompromised states [13-17]. Acyclovir treatment for patients diagnosed with EBV encephalitis is not recommended [18]. This recommendation is a category C-III, i.e., based on poor evidence, opinions of authorities, descriptive studies, and expert committee reports [18].

We present a case of EBV encephalitis in an adult male, who responded significantly to acyclovir treatment given at diagnosis.

## Case presentation

The patient, a 52-year-old African American man with HIV disease on HAART, was admitted with altered mental status (AMS) and weakness for three days' duration following a visit to his primary care physician’s office. He reported a three-week history of confusion and forgetfulness, having missed two days from work prior to presentation. These symptoms were preceded by several episodes of disorientation during his work commute. The patient reported a steady loss of weight over the previous 10 months despite compliance with his HAART medications.

He was diagnosed with HIV infection about 20 years prior and had a good response to treatment over the years. Other coexisting conditions included syphilis and hepatitis C, both successfully treated in the past. He had never been previously tested for EBV.

One year prior to presentation, he experienced a drop in his CD4 count owing to medication noncompliance due to insurance issues. However, his CD4 count was never recorded below 200 before admission. Approximately four months prior to his presenting illness, he had already resumed treatment with darunavir/cobicistat and emtricitabine/tenofovir alafenamide. His most recent CD4 count (four months prior to admission) was 383 x 10^9^/L, and HIV RNA was 40 copies/mL.

On admission, he was lethargic and dehydrated with orthostatic hypotension. He was disoriented to time and place. His tone and reflexes were normal, without meningism. His blood test results are shown in Table 1.

**Table 1:** Patient Blood Test Results BUN: blood urea nitrogen.

Blood Test	Results	Reference
Sodium, mEq/L	129	135–145
Potassium, mEq/L	3.4	3.5–5.1
BUN, mg/dL	14	7–25
Creatinine, mg/dL	1.42	0.6–1.2
Calcium, mg/dL	9.8	8.5–10.3
Hemoglobin, g/dL	16.3	14.6–17.8
WBC, × 10^9^/L	6.36	3.2–10.6
Neutrophils, %	37	38–80
Lymphocytes, %	46.2	11–49
Monocytes, %	15.1	4–12

His urine toxicology screen was negative, HIV RNA was 2030 copies/mL, and CD4 count was 187 x 10^9 ^cells/L. His blood and urine cultures were negative. CT chest showed right lower lobe pneumonia. Head CT without contrast showed no acute intracranial pathology.

He was started on IV piperacillin-tazobactam and vancomycin. He was rehydrated, and electrolyte abnormalities were corrected. However, by day four, he had become more confused and unable to walk. He developed an intention tremor and spiked a fever of 102°F (38.9°C) despite receiving broad-spectrum antibiotics. MRI brain with and without contrast was unremarkable for any acute intracranial pathology.

A lumbar puncture was done, and the patient was started empirically on IV penicillin G for possible neurosyphilis after serum RPR returned positive. As his mental status continued to worsen, IV acyclovir was initiated three days after starting penicillin G.

CSF analysis was suggestive of a viral infection (Table 2), and viral studies were positive only for EBV DNA by PCR in CSF (negative for cytomegalovirus, JC virus, West Nile virus, BK virus, and herpes simplex virus (HSV) 1 and 2). Cryptococcal antigen as well as culture for Cryptococcus were negative. Serum antibodies for West Nile virus, BK virus, and HSV 1 and 2 were also negative. Serum RPR was reactive at 1:1. Serum FTA-ABS was also reactive, as was the CSF VDRL. The patient became afebrile and started to improve after the initiation of acyclovir. A decision was therefore made to continue IV acyclovir for possible EBV encephalitis.

**Table 2:** CSF Analysis Results

CSF Studies	Results	Reference
Glucose, mg/dL	59	40–80
Protein, mg/dL	82	15–45
Lymphocyte, %	92	100
Monocyte, nm%	8	0
Neutrophil, %	0	0
RBC, cells/mm^3^	19	0–20
WBC, cells/mm^3^	100	0–5

After four days of acyclovir treatment, the patient experienced improvement in his memory as well as the intention tremor. He was able to walk by day seven of treatment. He was restarted on antiretroviral therapy and discharged to a subacute rehab facility. He completed an additional two weeks of IV acyclovir followed by four weeks of oral valacyclovir treatment with full recovery.

## Discussion

While ubiquitous, EBV is not commonly the sole culprit in symptomatic encephalitis; it is often found in association with other CNS infections or diseases such as malignant tumors [19,20]. This may be the basis of the IDSA’s recommendation against antiviral therapy when EBV DNA is found in CSF [18]. Our patient, however, was diagnosed after CSF exam came back positive for EBV DNA in the presence of AMS, intention tremor, and gait abnormalities. Similar symptoms were described by Zarlasht et al. in a case report of a Hispanic male who presented with paranoid behavior, complete loss of memory, generalized weakness, and urine incontinence and was diagnosed with EBV encephalitis [21]. Unlike that case, our patient had a positive CSF VDRL test which was deemed noncontributory to his presentation as he already had a history of syphilis that was adequately treated.

Findings of paraventricular lucencies and hyperintense lesions in multiple areas of the brain, including the basal ganglia, have been described on neuroimaging studies in patients with EBV encephalitis; however, there may be normal findings also, as was seen in our case [6,20,21].

Symptomatic management has been the mainstay in the treatment of EBV encephalitis, and most patients recover without any long-term sequelae [7]. However, rapidly progressive disease has been described, especially with significant immunosuppression [17]. Our patient started improving with IV acyclovir treatment and was back to his baseline mental status by day seven of treatment. Similar improvement was also described by Zarlasht et al. upon initiation of acyclovir [9,21]. Other etiologies (e.g., HIV encephalopathy or opportunistic infections) seem unlikely, given the acute presentation and rapid response to acyclovir. Given the patient’s clinical picture, CSF findings, and response to treatment, it is considered reasonable to attribute our patient’s neurologic symptoms to encephalitis caused by EBV infection.

Unlike opportunistic infections, EBV antigen levels do not correlate with the degree of immunosuppression, and levels were found to be highest among HIV-infected persons with CD4 counts between 100 and 400 [15]. Our patient fell within this category with a CD4 count of 187.

There is no recommended treatment for EBV encephalitis, and some patients have recovered spontaneously without treatment [7,18]. However, another study has shown that EBV encephalitis may be rapidly progressive, as seen in our patient, and can sometimes be fatal [17,22]. Clinicians should therefore consider EBV as an etiologic agent in HIV-positive patients on HAART who present with acute AMS. Management with antivirals early on may shorten their hospital course and prevent mortality.

## Conclusions

EBV encephalitis is more significant than has traditionally been described. Considering the currently available literature on the topic, a positive CSF EBV PCR test should not be overlooked, particularly in the setting of HIV, regardless of CD4 count. Prompt initiation of antiviral therapy such as acyclovir could be lifesaving in rapidly progressive EBV encephalitis. Since it may be difficult to determine who will go on to full recovery versus rapid deterioration, upon finding EBV in the CSF, in the appropriate clinical setting, it should be considered a possible causative agent rather than an innocent bystander.

## References

[1] Epstein MA, Achong BG, Barr YM (1964). Virus particles in cultured lymphoblasts from Burkitt’s lymphoma. Lancet.

[2] Tzellos S, Farrell PJ (2012). Epstein-barr virus sequence variation-biology and disease. Pathogens.

[3] Smatti MK, Al-Sadeq DW, Ali NH, Pintus G, Abou-Saleh H, Nasrallah GK (2018). Epstein-Barr virus epidemiology, serology, and genetic variability of LMP-1 oncogene among healthy population: an update. Front Oncol.

[4] Young LS, Rickinson AB (2004). Epstein-Barr virus: 40 years on. Nat Rev Cancer.

[5] Balfour HH Jr, Dunmire SK, Hogquist KA (2015). Infectious mononucleosis. Clin Transl Immunology.

[6] Hashemian S, Ashrafzadeh F, Akhondian J, Beiraghi Toosi M (2015). Epstein-barr virus encephalitis: a case report. Iran J Child Neurol.

[7] Khanal D, Singh T, Rabinstein A (2016). Epstein Barr virus encephalitis in adults: a case series. Neurology.

[8] Gilden DH, Mahalingam R, Cohrs RJ, Tyler KL (2007). Herpesvirus infections of the nervous system. Nat Clin Pract Neurol.

[9] Mahajan R, Anand KS, Juneja A, Garg J (2022). Epstein-Barr virus infection presenting as encephalitis in HIV - phenomenon not seen frequently. Indian J Sex Transm Dis AIDS.

[10] Wang J, Ozzard A, Nathan M, Atkins M, Nelson M, Gazzard B, Bower M (2007). The significance of Epstein-Barr virus detected in the cerebrospinal fluid of people with HIV infection. HIV Med.

[11] Fujimoto H, Asaoka K, Imaizumi T, Ayabe M, Shoji H, Kaji M (2003). Epstein-Barr virus infections of the central nervous system. Intern Med.

[12] Luzuriaga K, Sullivan JL (2010). Infectious mononucleosis. N Engl J Med.

[13] Dyachenko P, Smiianova O, Kurhanskaya V, Oleshko A, Dyachenko A (2018). Epstein-barr virus-associated encephalitis in a case-series of more than 40 patients. Wiad Lek.

[14] Weinberg A, Bloch KC, Li S, Tang YW, Palmer M, Tyler KL (2005). Dual infections of the central nervous system with Epstein-Barr virus. J Infect Dis.

[15] Trevillyan JM, Mahony AA, McLean C, Hoy JF (2013). Successful treatment of Epstein-Barr virus encephalitis in the setting of HIV-associated neurocognitive disorder: a diagnostic and therapeutic challenge. Antivir Ther.

[16] Sabat S, Agarwal A, Zacharia T, Labib S, Yousef J (2015). Epstein-Barr virus encephalitis presenting as cerebellar hemorrhage. Neuroradiol J.

[17] Polilli E, Sozio F, Mazzotta E (2010). Rapidly progressive and fatal EBV-related encephalitis in a patient with advanced HIV-1 infection at presentation: a case report and review of the literature. New Microbiol.

[18] Tunkel AR, Glaser CA, Bloch KC (2008). The management of encephalitis: clinical practice guidelines by the Infectious Diseases Society of America. Clin Infect Dis.

[19] Soldan SS, Lieberman PM (2020). Epstein-Barr virus infection in the development of neurological disorders. Drug Discov Today Dis Models.

[20] Soni N, Ora M, Singh R, Mehta P, Agarwal A, Bathla G (2023). Unpacking the CNS manifestations of Epstein-Barr virus: an imaging perspective. AJNR Am J Neuroradiol.

[21] Zarlasht F, Salehi M, Abu-Hishmeh M, Khan M (2017). Encephalitis treatment - a case report with long-term follow-up of EBV PCR in cerebrospinal fluid. Int J Gen Med.

[22] Huang L, Zhang X, Fang X (2021). Case report: Epstein-Barr virus encephalitis complicated with brain stem hemorrhage in an immune-competent adult. Front Immunol.

